# TNFα-Induced Oxidative Stress and Mitochondrial Dysfunction Alter Hypothalamic Neurogenesis and Promote Appetite Versus Satiety Neuropeptide Expression in Mice

**DOI:** 10.3390/brainsci12070900

**Published:** 2022-07-09

**Authors:** Mina Desai, Linsey Stiles, Adriana S. Torsoni, Marcio A. Torsoni, Orian S. Shirihai, Michael G. Ross

**Affiliations:** 1 The Lundquist Institute at Harbor-UCLA Medical Center, Torrance, CA 90502, USA; mikeross@ucla.edu; 2 Department of Obstetrics and Gynecology, David Geffen School of Medicine, University of California Los Angeles at Harbor-UCLA, Torrance, CA 90502, USA; 3 Department of Medicine, Endocrinology, David Geffen School of Medicine, University of California, Los Angeles, CA 90095, USA; lstiles@mednet.ucla.edu (L.S.); oshirihai@mednet.ucla.edu (O.S.S.); 4 Metabolism Theme, David Geffen School of Medicine, University of California, Los Angeles, CA 90095, USA; 5 Department of Molecular and Medical Pharmacology, University of California, Los Angeles, CA 90095, USA; 6 Laboratory of Metabolic Disorders, School of Applied Sciences, University of Campinas—UNICAMP, Limeira 13484-350, Brazil; atorsoni@unicamp.br (A.S.T.); torsoni@unicamp.br (M.A.T.); 7 Molecular Biology Institute, University of California, Los Angeles, CA 90095, USA; 8 Nutrition and Metabolism, Graduate Medical Sciences, Boston University School of Medicine, Boston, MA 02118, USA; 9 Department of Obstetrics and Gynecology, Charles R. Drew University, Los Angeles, CA 90059, USA

**Keywords:** Hypothalamic neuroprogenitor cells, reactive oxygen species, inflammation, proliferation, differentiation, maternal obesity, programmed hyperphagia

## Abstract

Maternal obesity results in programmed offspring hyperphagia and obesity. The increased offspring food intake is due in part to the preferential differentiation of hypothalamic neuroprogenitor cells (NPCs) to orexigenic (AgRP) vs. anorexigenic (POMC) neurons. The altered neurogenesis may involve hypothalamic bHLH (basic helix–loop–helix) neuroregulatory factors (Hes1, Mash1, and Ngn3). Whilst the underlying mechanism remains unclear, it is known that mitochondrial function is critical for neurogenesis and is impacted by proinflammatory cytokines such as TNFα. Obesity is associated with the activation of inflammation and oxidative stress pathways. In obese pregnancies, increased levels of TNFα are seen in maternal and cord blood, indicating increased fetal exposure. As TNFα influences neurogenesis and mitochondrial function, we tested the effects of TNFα and reactive oxidative species (ROS) hydrogen peroxide (H_2_O_2_) on hypothalamic NPC cultures from newborn mice. TNFα treatment impaired NPC mitochondrial function, increased ROS production and NPC proliferation, and decreased the protein expression of proneurogenic Mash1/Ngn3. Consistent with this, AgRP protein expression was increased and POMC was decreased. Notably, treatment with H_2_O_2_ produced similar effects as TNFα and also reduced the protein expression of antioxidant SIRT1. The inhibition of STAT3/NFκB prevented the effects of TNFα, suggesting that TNFα mediates its effects on NPCs via mitochondrial-induced oxidative stress that involves both signaling pathways.

## 1. Introduction

Maternal obesity during pregnancy is associated with macrosomic newborns [1] and offspring with childhood obesity and adult metabolic syndrome [2,3]. Animal models of maternal obesity have replicated evidence of human-programmed offspring hyperphagia and obesity [4,5]. The underlying cause of early onset offspring hyperphagia has been attributed to the altered development of the appetite regulatory site (hypothalamic arcuate nucleus, ARC) and neurogenesis [6,7]. The ARC contains at least two populations of neurons with opposing actions on food intake: primarily medial ARC orexigenic (NPY; neuropeptide Y and AgRP; agouti-related protein) and primarily lateral ARC anorexigenic (POMC; pro-opiomelanocortin and CART; cocaine- and amphetamine-regulated transcript) neurons. ARC development begins in fetal life during [8] which the neuroprogenitor cells (NPCs) in the peri-third ventricular zone undergo extensive proliferation, self-renewal, and terminal division into cells destined for neuronal or glial fates [9]. These neurons migrate to ultimate nuclei sites, differentiate to specific neuronal phenotypes, and form functional circuits. NPCs differentiated to neurons destined for the ARC further differentiate to orexigenic or anorexigenic phenotypes [10,11].

Early in development, the transmembrane Notch receptor involved in cell–cell communication regulates NPC proliferation by increasing the expression of Hes1, which suppresses proneurogenic bHLH factor expression (e.g., Mash1, Ngn3) [12]. Exposure to a maternal obesity/high-fat diet in utero results in altered fetal hypothalamic ARC development and programmed hyperphagia at birth [4]. The hyperphagia is due to the increased protein expression of orexigenic NPY/AgRP and reduced expression of anorexigenic POMC peptides. The neuronal counts show similar changes [7,13,14,15]. Consistent with the neuropeptide changes, the expression of neurogenic regulators (Ngn3 and Mash1, which promote POMC expression [16,17,18,19]) is decreased in the hypothalamic tissue and NPCs of newborns exposed to maternal obesity [6,7]. These findings suggest that developmentally altered neurogenesis is a significant factor contributing to neuronal phenotypes and offspring hyperphagia.

Whilst the underlying mechanism remains unclear, it is known that mitochondrial function is critical for neurogenesis [20] and is impacted by proinflammatory cytokines (TNFα) [21,22,23,24]. Notably, maternal obesity is associated with a proinflammatory and reactive oxidative stress environment [25,26]. Consistent with this, in obese pregnancies, increased levels of TNFα are seen in maternal and cord blood [27,28], indicating increased fetal exposure. Similarly in animal models of maternal obesity, elevated levels of TNFα are seen in maternal, fetal, and newborn plasma [29,30,31] with an increased expression of TNFα in the placenta and in brain regions including the hypothalamic ARC [32,33,34]. 

Collectively, the evidence provides a plausible mechanism of inflammatory mediators in the programmed neurogenesis and appetite in offspring exposed to maternal obesity. Hence, we tested the effects of exogenous TNFα in vitro on mitochondrial function and its downstream effect on the proliferation-, differentiation-, and appetite-regulating neuropeptides in the hypothalamic NPCs of newborns from normal mice pregnancies. As TNFα mediates its effects via STAT3 and NFκB signaling [35,36] and ROS production, we further studied the effects of inhibiting the individual signaling pathways and also verified the independent effects of ROS on hypothalamic NPCs, including the expression of SIRT1, which acts as an antioxidant [37].

## 2. Materials and Methods

### 2.1. Animals

Studies were approved by the Animal Research Committee of The Lundquist Institute at Harbor-University of California Los Angeles (formerly known as the Los Angles Biomedical Research Institute) and were in accordance with the American Association for Accreditation of Laboratory Care and National Institutes of Health guidelines. Four pregnant C57BL/6 mice (Jackson Laboratory, Bar Harbor, ME, USA) were housed at constant temperature and humidity on a controlled 12 h light/dark cycle with free access to food (LabDiet 5001, Brentwood, MO, USA) and drinking water. Following delivery, brains were collected from one-day-old newborn pups.

### 2.2. NPC Cultures

The hypothalamus was dissected from newborns, and three hypothalami from the same litter were pooled and placed in DMEM/F12 medium, trypsinized, and cultured as previously reported [38]. Briefly, NPCs were cultured in complete medium [NeurobasalTM Medium containing 1% anti-anti (Invitrogen, Waltham, MA, USA), 2% B27 (GIBCO, Cat#17504-044), 20 ng/mL FGF2 (Sigma, St. Louis, MI, USA), 20 ng/mL EGF (Sigma), 1 μg/mL heparin (Lilly, Indianapolis, IN, USA), and 2.5 μg/mL L-glutamine (Invitrogen)] or differentiation medium (cultured in absence of FGF2, EGF and heparin) and seeded in culture dishes pre-coated with 0.01% poly-L-lysine (Sigma).

### 2.3. NPC Mitochondrial Function

At day 7, cells cultured in complete medium were dissociated using TrypLE Express, seeded (40,000 cells/mL) on PDL-coated XF96 microplate, and treated with DMSO (control) or TNFα (20, 50, 100 pg/mL) [39] for 24 h (37 °C; 5% CO_2_). For mitochondrial respirometry measurements (Seahorse XF96 Extracellular Flux Analyzer (Agilent Technologies, Santa Clara, CA, USA), cells were washed with freshly prepared assay medium (Seahorse XF Base Medium supplemented with 10 mM glucose, 2 mM L-glutamine and 1 mM pyruvate; pH 7.4) and incubated at 37 °C without CO_2_ for 30 min at a volume of 175 µL/well. The oxygen consumption rate (OCR) and extracellular acidification rate (ECAR) were detected under basal conditions and after the sequential injection of inhibitors: ATP synthase inhibitor oligomycin (3 μM/well) was added to determine mitochondrial ATP production; electron transport chain uncoupler FCCP (carbonylcyanide p-trifluoromethoxy-phenylhydrazone, 1.8 μM/well) was added to determine the maximal respiration; and a mixture of rotenone and antimycin A (2 μM/well) was added to inhibit complexes I and III, respectively, to determine the spare capacity. This sequential process provided an estimation of the contribution of individual parameters for basal respiration, proton leak, maximal respiration, spare respiratory capacity, non-mitochondrial respiration, and ATP production [40].

### 2.4. In Vitro Treatment with TNFα and H_2_O_2_

Hypothalamic NPCs from one-day-old newborns were cultured in complete or differentiation medium [38] and treated with TNFα (5, 10, 20 pg/mL), H_2_O_2_ (0.5, 2.5, 10 µM), or DMSO (untreated control cells) for 24 h. NPCs treated in complete medium were used to measure the ROS (fluorescence assay), proliferation index (MTT assay, Sigma) [41,42], and protein expression of the NPC marker (Nestin), activated notch (ICD), neuroproliferative bHLH factor (Hes1), and antioxidant (SIRT1) by Western blot. NPCs treated in differentiation medium were used to measure protein expression of proneurogenic transcription factors (Mash1, Ngn3) and neuropeptides (AgRP, POMC).

### 2.5. In Vitro Treatment with Inhibitors

Hypothalamic NPCs from one-day-old newborns were cultured in complete or differentiation medium and treated with inhibitors of STAT3 signaling (5 µM AG490; JAK2/STAT3 inhibitor [8]) or NFκB activation (5 µM TPCA1; IKK-2 inhibitor [43]) in presence or absence of TNFα (10 pg/mL) for 24 h. NPC proliferation index (MTT assay) and the protein expression of STAT3, pSTAT3, NFĸB, pNFĸB, Hes1, Mash1, and POMC were analyzed by Western blot.

### 2.6. ROS Assay

Reactive oxygen species levels were measured using commercial Assay Kit (ab113851, abcam, Waltham, MA, USA) at Ex/Em: 485/535.

### 2.7. Proliferation Assay

NPC proliferation rate was determined by the MTT (3-(4,5-dimethylthiazol-2-yl)-diphenyl tetrazolium bromide; Sigma) colorimetric assay. MTT solution (5 mg/mL in BPS) was added to each cell culture well and incubated for 1 h at 37 °C in CO2 incubator. The cultured cells were harvested, and the MTT reaction product formazan was extracted with acidic isopropanol (isopropanol in 0.04N HCl). The optical density of the formazan solution was measured on an ELISA plate reader (VICTOR™ 1420 Multilabel Counter) at 570 nm. The cell proliferating index was expressed as a value of OD 570 nm.

### 2.8. Western Blot

For protein expression analysis, the disassociated neurosphere cells in complete or differentiating medium were harvested and dissolved in RIPA solution (Cell Signaling, Beverly, MA, USA) with a protease inhibitor cocktail (Thermo Fisher Scientific, Irwindale, CA, USA); briefly sonicated and the cell lysates processed for the determination of protein content by BAC^TM^ Protein Assay Kit (Thermo Fisher Scientific). Westerns were performed as previously reported by our group [38]. For the detection of pSTAT3 and NFκBp65, NaF (50 mM) was added to all buffers, which had been demonstrated to be an effective phosphoseryl and phosphothreonyl protein phosphatase inhibitor. Antibodies were obtained from Santa Cruz Biotechnology, Inc (Santa Cruz, CA, USA) unless otherwise specified: AgRP (1:500, 14 kDA, sc-50299); POMC (1:500, 30 kDa, sc-20148); Hes1 (1:500, 35 kDa, sc-25392); Mash1 (1:500, 30 kDa, sc-13222); Ngn3 (1:1000, 23 kDa, ab38548, Abcam, Cambridge, MA); SIRT1 (1:2000, 120 kDa, sc-5322), Cleaved NOTCH1 (ICD, 1:1000, 100 kDa, Cell Signaling), pSTAT3 (1:1000; 92 KD Thermo Fisher); and pNFκB (1:1000, 65kd, NB100-2176, Novus Biologicals, Littleton, CO, USA). The secondary antibody included anti-mouse IgG-HRP (Cell Signaling), anti-rabbit IgG-HRP (Cell Signaling), and anti-goat IgG-HRP (Santa Cruz Biotechnology, Inc., Santa Cruz, CA, USA). The blots were applied with SuperSignal West Pico Chemiluminescence Substrate (Pierce, Rockford, IL, USA) to produce chemiluminescence, which was visualized by exposing blots to x-ray film (HyBlot CL Autoradiography Film; Denville Scientific, Inc., Metuchen, NJ, USA). The total protein was used as the loading control. STAT3, pSTAT3, NFκB, and pNFκB were normalized to the loading protein and the ratios of pSTAT3/STAT3 and pNFκB/ NFκB determined.

### 2.9. Data Analysis

NPCs were established from *n* = 4 independent animals, and each treatment in vitro was undertaken in duplicate.

OCR was measured before and after the addition of inhibitors to derive several parameters of mitochondrial respiration that included basal respiration (derived by subtracting non-mitochondrial respiration from baseline cellular OCR), ATP-linked respiration (derived by subtracting the oligomycin rate from baseline cellular OCR), and maximal respiratory capacity (derived by subtracting non-mitochondrial respiration from the FCCP rate).

ANOVA with Dunnett’s post-hoc was used to compare treated versus non-treated NPCs and TNFα versus TNFα+inhibitor effects. Values are presented as fold change (Mean ± SEM).

## 3. Results

### 3.1. TNFα Impairs NPC Mitochondrial Function, Promotes ROS Production, and Alters Neuropeptide Expression

**Figure 1:**
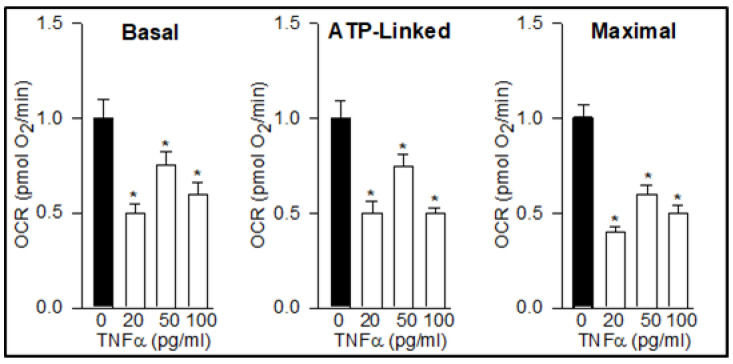
TNFα Impairs NPC Mitochondrial Function. Hypothalamic NPCs were treated with various doses (20, 50, and 100 pg/mL) of TNFα in complete medium for 24 h. Basal, ATP-linked, and maximal respiration were determined. Values are normalized to untreated control cells and expressed as fold change (Mean ± SEM of *n* = 4 independent experiments with each treatment performed in duplicate). * *p*< 0.05 vs. untreated cells.

The treatment of hypothalamic NPCs with TNFα for 24 h impaired mitochondrial function, as evidenced by reduced basal, ATP-linked, and maximal OCR at all three doses with ~50% reduction at doses 20 and 100 pg/mL (Figure 1).

**Figure 2:**
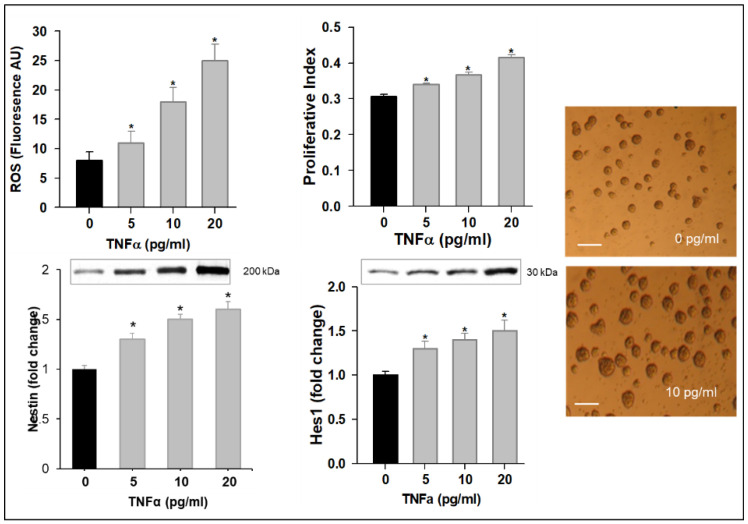
TNFα Increases NPC ROS and Promotes Proliferation. Hypothalamic NPCs were treated with various doses (5, 10, and 20 pg/mL) of TNFα in complete medium for 24 h. Values are (Mean ± SEM of *n* = 4 independent experiments with each treatment performed in duplicate) normalized to untreated control cells and expressed as fold change. For protein expression, representative immunoblots are shown. Images of untreated (0 pg/mL) and TNFα-treated (10 pg/mL) cells (×20; scale bar = 50μm). * *p* < 0.05 vs. untreated cells.

**Figure 3:**
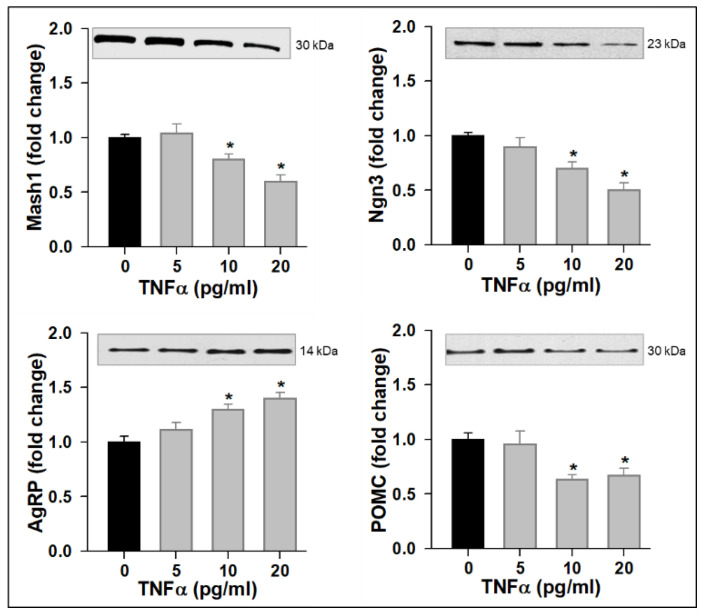
TNFα Suppresses Proneurogenic bHLH Genes and Alters Neuropeptide Expression. Hypothalamic NPCs were treated with various doses (5, 10, 20 pg/mL) of TNFα in differentiation medium for 24 h. Values are (Mean ± SEM of *n* = 4 independent experiments with each treatment performed in duplicate) normalized to untreated control cells and expressed as fold change. For protein expression, representative immunoblots are shown.* *p* < 0.05 vs. untreated cells.

In view of the 50% reduction in mitochondrial function at 20 pg/mL TNFα, our subsequent studies used lower doses of TNFα (≤20 pg/mL). With increasing TNFα doses in complete medium, NPCs exhibited increased levels of ROS with an increased expression in Hes1. Consistent with this, there was a dose-dependent increase in proliferation and Nestin expression (Figure 2). However, in differentiation medium, TNFα treatment (10 and 20 pg/mL) resulted in the decreased protein expression of Mash1 and Ngn3 with a corresponding increased AgRP and decreased POMC (Figure 3).

### 3.2. ROS Suppresses Antioxidant SIRT1 and Alters NPC Proliferation/Differentiation and Neuropeptide Expression

**Figure 4:**
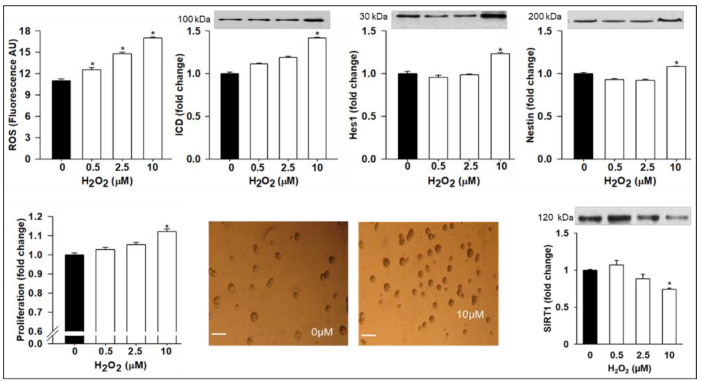
H_2_O_2_ Promotes ROS Production and Upregulates NPC Notch/Hes1 Pathway and NPC Proliferation with Downregulation of SIRT1. Hypothalamic NPCs were treated with various doses (0.5, 2.5, and 10 µM) of H_2_O_2_ in complete medium for 24 h. Values are (Mean ± SEM of *n* = 4 independent experiments with each treatment performed in duplicate) normalized to untreated control cells and expressed as fold change. For protein expression, representative immunoblots are shown. Images of untreated (0 µM) and H_2_O_2_ treated (10 µM) cells (×20; scale bar = 50 μm). * *p* < 0.05 vs. untreated cells.

**Figure 5:**
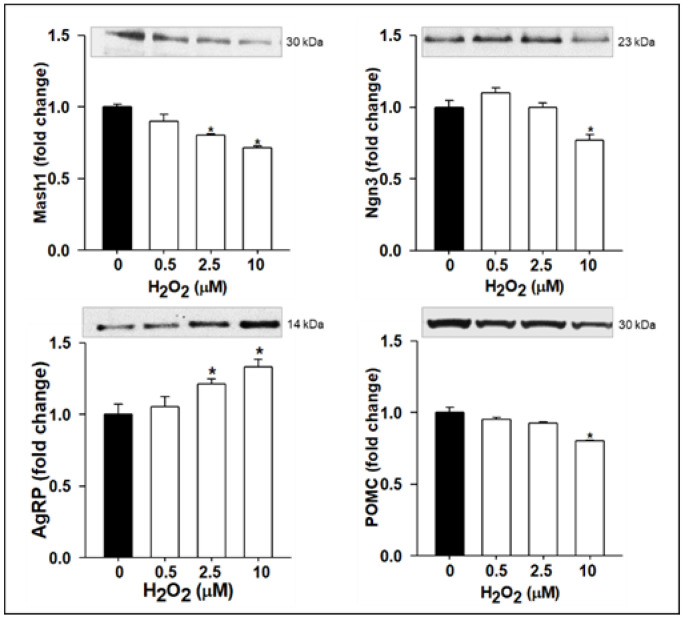
H_2_O_2_ Reduces NPC Proneurogenic bHLH Genes and Alters Neuropeptide Expression. Hypothalamic NPCs were treated with various doses (0.5, 2.5, and 10 µM) of H_2_O_2_ in differentiation medium for 24 h. Values are (Mean ± SEM of *n* = 4 independent experiments with each treatment performed in duplicate) normalized to untreated control cells and expressed as fold change. For protein expression, representative immunoblots are shown. * *p* < 0.05 vs. untreated cells.

To elucidate whether the TNFα-induced effects on neurogenesis were mediated via increased ROS levels, we measured direct effects of ROS on NPCs. Treatment with H_2_O_2_ doses in the complete medium showed a quantitative increase in NPC ROS levels with a concomitant increased protein expression of activated Notch 1 (ICD), Hes1 (10 µM H_2_O_2_), and Nestin. In parallel, NPCs proliferation was increased. However, the SIRT1 protein expression was decreased (Figure 4). In differentiation medium, treatment with TNFα resulted in the reduced protein expression of Mash1 and Ngn3 with decreased POMC and increased AgRP (Figure 5).

### 3.3. Inhibition of TNFα-Mediated STAT3/NFκB Signaling Normalizes NPC Proliferation/Differentiation

**Figure 6:**
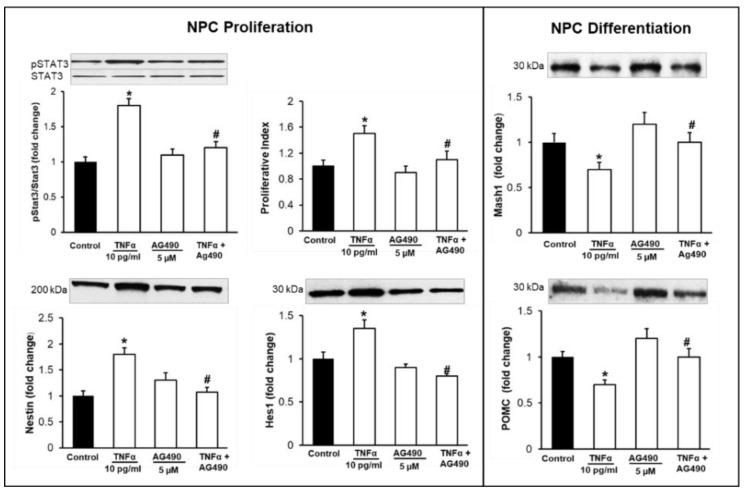
Effects of AG490 Inhibitor on pSTAT3 and NPC Proliferation/Differentiation. Hypothalamic NPCs were treated with TNFα (10 pg/mL), AG490 (5 µM), and TNFα + inhibitor in complete or differentiation medium for 24 h. Values are (Mean ± SEM of *n* = 4 independent experiments with each treatment performed in duplicate) normalized to untreated control cells and expressed as fold change. * *p* < 0.01 vs. untreated cells; # *p* < 0.05 TNFα + AG490 vs. TNFα.

**Figure 7:**
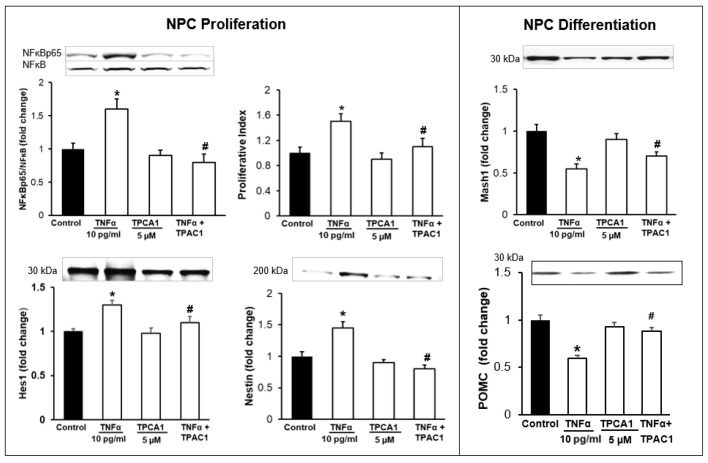
Effects of NFĸB Inhibitor (TPCA1) on NPC Proliferation/Differentiation. Hypothalamic NPCs were treated with TNFα (10 pg/mL), TPCA1 (5 µM) and TNFα + inhibitor in complete or differentiation medium for 24 h. Values are (Mean ± SEM of *n* = 4 independent experiments with each treatment performed in duplicate) normalized to untreated control cells and expressed as fold change. * *p* < 0.05 vs. untreated cells; # *p* < 0.05 TNFα + TPAC1 vs. TNFα.

Treatment with AG490 inhibitor significantly inhibited the expression of pSTAT3 and prevented the effects of TNFα. That is, it normalized NPC proliferation and the protein expression of Hes1, Mash1, and POMC (Figure 6). Similarly, treatment with TPAC1 inhibitor significantly inhibited the expression of NFκBp65 and normalized NPC proliferation and the protein expressions of Hes1, Mash1, and POMC (Figure 7). There was no significant change in the protein expressions of STAT3 and NFκB.

## 4. Discussion

Previous studies have shown that TNFα negatively regulates embryonic and adult neurogenesis [44,45,46]. However, to our knowledge, this is the first study to demonstrate the effects of TNFα on hypothalamic NPCs from one-day-old newborns and provide a plausible pathway of TNFα-mediated mitochondrial dysfunction effects on NPC proliferation/differentiation with resultant altered appetite/satiety neuropeptide expression. We speculate that this may be a potential mechanistic pathway contributing to the programmed hyperphagia in the offspring of obese dams.

TNFα effects are primarily mediated via its receptors (TNFR1 and TNFR2), both of which are expressed in the hypothalamus [47,48] and NPCs [44,49]. TNFα binding to the receptors activate both canonical NFκB and STAT3 signaling pathways [35,36]. Briefly, NFκB proteins are comprised of transcription factors that remain inactive in the cytosol bound to inhibitor IκB proteins. TNFα-induced phosphorylation of IκB by the IκB kinase complex (IKKβ, IKKα, and NEMO) leads to IκB degradation, the nuclear translocation of NFκBp65, and induction of transcription of target genes [36]. In response to TNFα, STAT3 is phosphorylated by receptor-associated Janus kinases (JAK) and translocated to the nucleus where they act as transcription activators [35]. The activations of both NFκB and STAT3 pathways lead to mitochondrial dysfunction [50,51,52] and increased oxidative stress [53,54]. It is known that mitochondrial function is critical for neurogenesis [55], with mitochondrial apoptosis and ROS levels modulating NPC proliferation [56,57] and differentiation [58,59,60].

Complementing mitochondrial effects, NPC proliferation and differentiation processes are regulated by a spatial/temporal interplay of pathways, including cell communication factors (e.g., Notch/Hes1) and a series of neuroregulatory bHLH transcription factors, including Mash1 and Ngn3, among others. Early in development, the transmembrane Notch receptor involved in cell–cell communication regulates NPC proliferation by increasing the expression of Hes1, which suppresses proneurogenic bHLH factor expression (e.g., Mash1, Ngn3). Notably, both Ngn3 and Mash1 are required for the normal development of POMC neurons [18], while Ngn3 also inhibits NPY expression [19]. Thus, Ngn3(-/-) mice express markedly reduced POMC [19] and an increased number of NPY cells [16].

In the present study, treatment of hypothalamic NPCs with TNFα in complete medium resulted in impaired mitochondrial function as evident by the lower basal, ATP-linked and maximal oxygen consumption rate. Furthermore, TNFα treatment increased ROS levels with a corresponding increased expression of Hes1 and augmented the proliferation of NPCs. Treatment in differentiation medium resulted in suppressed Mash/Ngn3 with increased AgRP and decreased POMC expression. These findings are consistent with our postulated mechanism of TNFα-mediated programmed hyperphagia in offspring of maternal obesity/high-fat diets.

To further verify that TNFα likely mediates its effects on NPCs via induced oxidative stress, we studied the direct effects of H_2_O_2_ on NPCs and showed increased ROS levels with increased NPC proliferation and similar changes in bHLH genes and neuropeptides akin to TNFα. Although NPCs have lower levels of ROS than differentiated cells [35], as a result of the higher expression of uncoupling protein 2 (UCP2) and glutathione peroxidase (GPx) [59,61], previous studies show that ROS can increase Notch/Hes1 expression [62] and NPC proliferation [56,57] and also modulate NPC differentiation [58,59,60].

As ROS are generated mainly as by-products of mitochondrial respiration, mitochondria are the primary target of oxidative damage and dysfunction. The imbalance between mitochondrial ROS production and removal due to the overproduction of ROS and/or decreased antioxidants defense activity results in oxidative stress [63,64]. SIRT1, an NAD+-dependent protein deacetylase, has been shown to modulate the regulation of a variety of cellular processes associated with ROS including neurogenesis [65,66]. It is metabolically active in the hypothalamus and is inhibited by increased levels of ROS [67]. Through its deacetylation activity, it suppresses NFκB signaling and Hes1 [37]. This is consistent with our results of ROS-mediated decreased SIRT1 and increased Hes1.

The inhibition of signaling pathways STAT3 by AG490 [68] and NFκB by TPAC1 [69] prevented TNFα-mediated effects on NPCs. Although TPCA1 is considered a potent and selective inhibitor of IκB kinases [69], recent studies indicate that TPCA1 may be a dual inhibitor of STAT3 and NFκB [70]. This may in part explain our comparable findings on inhibiting STAT3 and NFκB pathways, suggesting that both pathways are important for NPC proliferation/differentiation or, alternatively, that the STAT3 pathway may be of greater importance. As NFκB and STAT3 are both cytosolic [56,71] and mitochondrial [72,73], the activation of these factors by TNFα may have direct nuclear effects on bHLH gene expression or are mediated via induced mitochondrial dysfunction [21,22,23]. Notably, STAT3 directly interacts with Mash1 and Ngn3 [35].

TNFα and other pro-inflammatory cytokines play important roles in the control of body energy stores. TNFα levels are correlated with maternal BMI and significantly increased in obese [74] and gestational diabetic women [75], resulting in fetal exposure to proinflammatory cytokines. In obese pregnant women, placental TNFα expression is increased [76] with higher levels of amniotic fluid [74] and umbilical cord blood TNFα [27,28]. Accordingly, cord blood TNFα levels are two-fold higher in newborns of overweight/obese mothers and are positively correlated with maternal BMIs [27]. In rodents, a high-fat diet significantly increases serum TNFα in both obese and obesity-resistant rats [77], and neonatal overfeeding markedly increases mouse serum TNFα [78]. Diet-induced obese murine models evidence increased inflammation [33] and impaired brain mitochondrial function, as high-fat diet induces increased hypothalamic TNFα mRNA prior to substantial weight gain and peripheral inflammation. Further, sustained high-fat diet results in a 25% reduction in POMC cells [79], suggesting that inflammatory-mediated impaired satiety contributes to obesity.

Other cytokines, such as IL6 and IL1B [80,81] and saturated fatty acids (e.g., palmitate) [82,83], also increase ROS and induce mitochondrial dysfunction. Notably, maternal obesity is associated with reactive oxidative stress and a lipotoxic environment [25,26] and, consistent with this, in obese pregnancies, increased levels of IL6 and palmitate are seen in maternal and cord blood [84,85,86]. The in vitro treatment of rodent NPCs with varying doses of IL6 [87], IL1B [88], and palmitate [89,90] inhibits neurogenesis at higher doses, likely via STAT3 activation [87,90]. In addition to these, an array of other factors including hormones (leptin and insulin) [38], β-catenin, and growth factors (IGF1, FGF) influence neurogenesis by promoting NPC proliferation and determining neuronal versus glial fate [91,92,93].

Various natural and synthetic antioxidants have been shown to have beneficial effects in reducing ROS and inflammation. For example, N-acetyl-l-cysteine (NAC) suppresses TNF-induced NFκB activation through the inhibition of IκB kinases, and as it effectively crosses the blood-brain barrier [94], it also reduces brain ROS [95,96]. More effective antioxidants such as Mito-TEMPO specifically target and accumulate in the mitochondria. It acts as a mitochondrial superoxide scavenger [97] and prevents cell death in vitro [98] and oxidative stress in vivo [99]. Several synthetic mitochondria-targeted ROS scavengers, such as MitoVit-E (vitamin E covalently attached to a triphenylphosphonium cation), are also effective as they easily pass through cell membranes, including the blood–brain barrier, into cells and tissues affected by mitochondria ROS [64]. Lastly, SIRT1 (antioxidant and histone deacetylase) inhibits NFκB signaling and also binds to the promoters of TNFα and IL1β and, through deacetylation, suppresses the expression of these cytokines.

## 5. Conclusions

In summary, the current study specifically shows that TNFα impairs NPCs mitochondrial function, increases ROS production leading to activation of Notch/Hes1, and promotes NPC proliferation. Consistent with this, the proneurogenic factors Mash1/Ngn3 are suppressed causing an increase in AgRP and decrease in POMC expression. Notably, treatment with H_2_O_2_ produced similar effects as TNFα and reduced antioxidant SIRT1. The inhibition of STAT3/NFκB signaling prevented the effects of TNFα, suggesting that TNFα mediates its effects on NPCs likely involving both signaling pathways. Nonetheless, future studies that directly test the roles of TNFα, mitochondrial dysfunction, reactive oxygen species, and changes in protein expression profile are needed to confirm this pathway and its role in neurogenesis and hyperphagia.

## Data Availability

Not applicable.
