# Correction: Desai et al. TNFα-Induced Oxidative Stress and Mitochondrial Dysfunction Alter Hypothalamic Neurogenesis and Promote Appetite Versus Satiety Neuropeptide Expression in Mice. *Brain Sci.* **2022**, *12*, 900

**DOI:** 10.3390/brainsci15111153

**Published:** 2025-10-28

**Authors:** Mina Desai, Linsey Stiles, Adriana S. Torsoni, Marcio A. Torsoni, Orian S. Shirihai, Michael G. Ross

**Affiliations:** 1 The Lundquist Institute at Harbor-UCLA Medical Center, Torrance, CA 90502, USA; mikeross@ucla.edu; 2 Department of Obstetrics and Gynecology, David Geffen School of Medicine, University of California Los Angeles at Harbor-UCLA, Torrance, CA 90502, USA; 3 Department of Medicine, Endocrinology, David Geffen School of Medicine, University of California, Los Angeles, CA 90095, USA; lstiles@mednet.ucla.edu (L.S.); oshirihai@mednet.ucla.edu (O.S.S.); 4 Metabolism Theme, David Geffen School of Medicine, University of California, Los Angeles, CA 90095, USA; 5 Department of Molecular and Medical Pharmacology, University of California, Los Angeles, CA 90095, USA; 6 Laboratory of Metabolic Disorders, School of Applied Sciences, University of Campinas—UNICAMP, Limeira 13484-350, Brazil; atorsoni@unicamp.br (A.S.T.); torsoni@unicamp.br (M.A.T.); 7 Molecular Biology Institute, University of California, Los Angeles, CA 90095, USA; 8 Nutrition and Metabolism, Graduate Medical Sciences, Boston University School of Medicine, Boston, MA 02118, USA; 9 Department of Obstetrics and Gynecology, Charles R. Drew University, Los Angeles, CA 90059, USA

In the original publication [1], there was a mistake in Figure 7. The authors had erroneously pasted the Hes1 bar graph for the POMC bar graph in Figure 7. The corrected Figure 7 appears below. The authors state that the scientific conclusions are unaffected. This correction was approved by the Academic Editor. The original publication has also been updated.

## Figures and Tables

**Figure 7:**
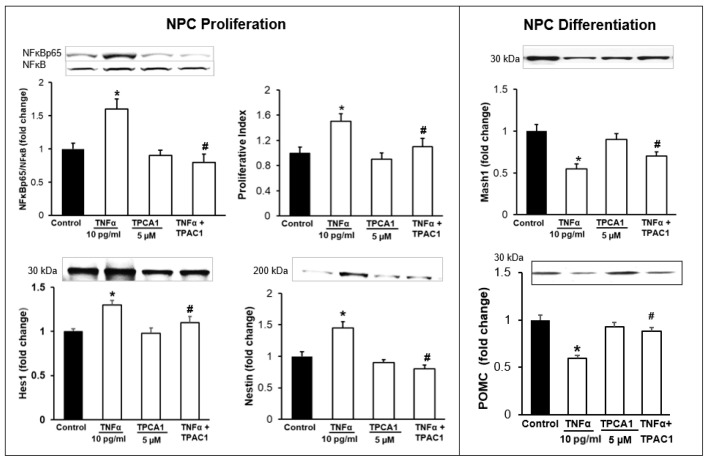
Effects of NFĸB Inhibitor (TPCA1) on NPC Proliferation/Differentiation. Hypothalamic NPCs were treated with TNFα (10 pg/mL), TPCA1 (5 µM) and TNFα + inhibitor in complete or differentiation medium for 24 h. Values are (Mean ± SEM of *n* = 4 independent experiments with each treatment performed in duplicate) normalized to untreated control cells and expressed as fold change. * *p* < 0.05 vs. untreated cells; # *p* < 0.05 TNFα + TPAC1 vs. TNFα.
